# Psychological Traits of Bariatric Surgery Candidates and Predictors of Outcomes

**DOI:** 10.3390/jpm15060215

**Published:** 2025-05-26

**Authors:** Abed Hadipour Lakmehsari, Carmela Mento, Claudia Scaramuzzino, Federica Arena, Fabrizio Turiaco, Maria Rosaria Anna Muscatello, Giuseppe Navarra, Gianluca Pandolfo, Clara Lombardo

**Affiliations:** 1 Department of Cognitive, Psychological, Pedagogical Sciences and Cultural Studies, University of Messina, 98122 Messina, Italy; abed.hadipourlakmehsari@unime.it; 2 Department of Biomedical and Dental Sciences and Morphofunctional Imaging, University of Messina, 98122 Messina, Italy; fturiaco@unime.it (F.T.); mmuscatello@unime.it (M.R.A.M.); gpandolfo@unime.it (G.P.); 3 Department “Scienze della Salute”, University of Catanzaro, 88100 Catanzaro, Italy; claudia.scaramuzzino@unicz.it (C.S.); clara.lombardo@unicz.it (C.L.); 4 Psychiatric Unit, Polyclinic Hospital, 98122 Messina, Italy; farena@unime.it; 5 Department of Adult and Development Age Human Pathology “Gaetano Barresi”, University of Messina, 98122 Messina, Italy; gnavarra@unime.it

**Keywords:** bariatric surgery, body image, predictors, obesity, personalised medicine

## Abstract

**Background:** Obesity is associated with a reduced life expectancy of 5 to 20 years, depending on the severity of the condition and the presence of comorbidities. Beyond first- and second-line interventions such as lifestyle changes, pharmacotherapy, which includes appetite suppressants, drugs that reduce fat absorption or regulate neurohormonal pathways, and endoscopic procedures, bariatric surgery is currently considered one of the most effective long-term interventions for severe obesity. This exploratory study investigates the psychological functioning of bariatric surgery candidates in the preoperative phase, aiming to identify risk factors and potential predictors of response to surgery in an Italian sample. **Methods:** This is a retrospective, observational study with follow-up. Participants, evaluated between September 2021 and September 2022 at Messina University Hospital, were recontacted approximately one year after surgery for re-evaluation. Of the 97 initial patients, 33 agreed to complete online questionnaires for follow-up. **Results**: The baseline data showed no significant differences between men and women in psychological assessments. In the subgroup that completed the follow-up, significant changes were observed, including a reduction in BMI and an increase in the discomfort index (Body Uneasiness Test) post-surgery, with large effect sizes in both cases. However, despite these changes, the regression analysis revealed that preoperative BMI values were not directly related to postoperative body image difficulties. These findings suggest a limited psychological impact of bariatric surgery, emphasizing the need for tailored psychological interventions to address these issues. **Conclusions**: While the intervention confirmed its effectiveness in reducing BMI, improvements in psychological well-being were less pronounced. In particular, a significant increase in body image concerns (PSDI) emerged after surgery, suggesting the need to address body-related distress in post-surgical care. These findings may suggest multidisciplinary approaches that integrate physical and psychological interventions may be needed to maximise long-term benefits. Further research should explore strategies to enhance patient awareness of treatment options, body image issues, and potential complications. These results should be interpreted with caution considering the limitations associated with this study such as a small sample size, lack of a control group, and the use of self-report and online methods to gather data, among others.

## 1. Introduction

Non-communicable diseases (NCDs)—such as cardiovascular disease, cancer, and diabetes mellitus—represent the leading cause of premature mortality worldwide, accounting for over 70% of global deaths according to the World Health Organization (WHO) [1]. Obesity is a major risk factor for these conditions and is associated with a significant reduction in life expectancy, which can vary from 5 to 20 years depending on disease severity and the presence of comorbidities [1,2,3].

Obesity is defined as an excessive accumulation of body fat that compromises health and is clinically diagnosed when the body mass index (BMI) exceeds 30 kg/m^2^ [3]. Individuals with obesity are at increased risk for a range of medical conditions, including type 2 diabetes, non-alcoholic fatty liver disease, cardiovascular disease, musculoskeletal disorders, certain types of cancer (e.g., breast, ovarian, liver, kidney, colon), and neurodegenerative diseases such as Alzheimer’s disease [4,5,6].

Beyond physical health, obesity is also associated with significant psychiatric comorbidities, including depression, anxiety disorders, binge eating disorder, and an impaired quality of life [4]. In addition, obesity can negatively impact on social functioning, employment, and productivity, often contributing to social stigma and discrimination. Importantly, the World Obesity Federation and other international institutions now consider obesity not merely a risk factor for other diseases but a chronic and progressive disease in itself, characterised by complex interactions between genetic, metabolic, psychological, and environmental factors [4,5].

From a pathophysiological perspective, obesity alters homeostatic mechanisms regulating energy balance, making weight loss difficult to achieve and sustain over time [5]. Biological processes—such as increased appetite, reduced energy expenditure, and metabolic adaptations—interact with psychological factors, including emotional dysregulation, stress, and maladaptive coping strategies, contributing to overeating and weight regain [6].

Although a balanced diet and regular physical activity are essential preventive strategies, these measures alone often prove insufficient for individuals with severe obesity [7]. Indeed, behavioural interventions frequently produce only temporary weight loss, with high rates of weight regain following treatment’s discontinuation [8]. Pharmacological approaches are considered either for individuals with a BMI ≥ 27 kg/m^2^ with at least one obesity-related comorbidity and those with a BMI ≥ 30 kg/m^2^. Anti-obesity medications (AOMs), which include appetite suppressants and drugs that reduce fat absorption or regulate neurohormonal pathways, with the growing class of nutrient-stimulated hormone-based (NUSH) medications, still deserve further evaluation in terms of short- and long-term efficacy, safety and tolerability, and cost-effectiveness issues [9].

Increasing attention has therefore been devoted to the role of psychological functioning in the development and maintenance of obesity. In particular, emotional eating—the tendency to consume food in response to negative emotions—has been identified as a maladaptive coping mechanism that may hinder weight management efforts [10].

Depressive symptoms are frequently observed in individuals affected by obesity, with studies indicating a prevalence reaching up to 30% when compared to age-matched individuals in the general population [7]. Several investigations have highlighted cognitive impairments in people with obesity relative to those of normal weight, particularly in areas such as memory performance, irrespective of age [8]. In addition, deficits in executive functions—such as planning, problem-solving, cognitive flexibility, and inhibitory control—have been documented, potentially indicating dysfunctions involving frontal lobe activity [11]. Obesity has also been associated with the emergence of cognitive impairments typically linked to ageing processes [12,13,14]. Although establishing a direct causal link between obesity and neuropsychiatric disorders remains challenging in clinical contexts, improvements in mood and cognitive functioning following significant weight loss—either through dietary changes or bariatric surgery—lend support to the notion that obesity may exert a negative influence on neuropsychological health [15,16,17,18]. Conversely, several findings suggest that cognitive and emotional disturbances may precede and contribute to the development of obesity, pointing to a possible bidirectional association between obesity and neuropsychiatric conditions [4,19,20].

To date, bariatric surgery remains the most widely adopted intervention for the treatment of severe obesity, particularly in individuals presenting with comorbid type 2 diabetes [21].

In 2018, approximately 252,000 bariatric surgeries were performed in the United States [22].

The main clinical benefits of bariatric surgery include a significant and often rapid reduction in body weight, improvement in obesity-related conditions (such as type 2 diabetes, hypertension, and sleep apnoea), enhanced quality of life, and increased life expectancy. However, although short-term outcomes are generally favourable, these results should be interpreted cautiously in light of possible surgical risks and the growing evidence of suboptimal long-term efficacy for a substantial proportion of patients [23,24]. The available data indicate that postoperative complications, both early and late, are less frequent than previously estimated, with an incidence of serious adverse events in the early postoperative phase of approximately 4.3% [22].

Importantly, despite the initial success of bariatric surgery in promoting weight loss and improving metabolic outcomes, several studies have highlighted the risk of weight regain over time and the persistence or emergence of psychological distress [25,26]. Long-term follow-up studies have shown that 20% to 30% of patients may experience significant weight regain within 5 to 10 years after surgery [26], often associated with maladaptive eating behaviours, insufficient lifestyle changes, and unresolved psychological vulnerabilities [7,10]

This exploratory study aims to examine the psychological functioning of bariatric surgery candidates in the preoperative phase, identifying risk factors and possible predictors of response to surgery in an Italian sample.

## 2. Materials and Methods

### 2.1. Study Design

This was a retrospective and naturalistic study with a small follow-up sample, which reflects real-world clinical practice but also implies some inherent limitations in terms of causal inferences made from our data. Therefore, one needs to consider the exploratory nature of this work when considering the results and their interpretations. Candidates for bariatric surgery, assessed between September 2021 and September 2022 at the Messina University Hospital, who had undergone surgery and whose information were available, were included in the study. These patients were re-contacted for reassessment approximately one year after surgery. Of the 97 patients contacted, 33 agreed to complete the online questionnaires. It is noteworthy that only 9 patients in the baseline sample of 97 declared that they took psychotropic drugs at the time of the first interview. Out of these 9, only 2 responded to the invitation for the follow-up and thus were included in the follow-up sample of 33 participants. However, no information about their drug regimen was available at the second assessment.

### 2.2. Psychological Assessments

#### 2.2.1. Beck Depression Inventory (BDI-II)

The Beck Depression Inventory—second edition (BDI-II) [27] is a 21-item self-assessment questionnaire designed to assess the intensity of depressive symptoms in adolescents and adults. Participants must select the statement that best describes their emotional state over the past two weeks, including that day. Each item is rated on a scale from 0 to 3, and the total score is obtained by summing the scores of all the items. A score between 0 and 13 indicates minimal levels of depression, between 14 and 19 suggests mild depression, and between 20 and 28 indicates moderate depression, while scores between 29 and 63 are associated with severe depression [28].

#### 2.2.2. Binge Eating Scale (BES)

The Binge Eating Scale (BES) is a 16-item self-assessment questionnaire designed to measure the behavioural, emotional and cognitive aspects of binge eating [29]. Each item includes three or four statements describing a specific characteristic of the binge eating episode (e.g., binge eating), with options varying in severity. For example, one of the statements may indicate no difficulty in stopping when full, while another may reflect greater difficulty in controlling food intake, even to the point of resorting to compensatory behaviour. Participants should choose the statement that best represents their experience. Higher scores on the BES indicate a greater severity of binge eating [30].

#### 2.2.3. Body Uneasiness Test (BUT)

The Body Uneasiness Test (BUT) is a self-assessment questionnaire designed to investigate several areas related to body image in both clinical and non-clinical populations [31]. It examines dissatisfaction with body shape and/or body weight, avoidance, compulsive controlling behaviour, and feelings of detachment and estrangement from one’s own body, as well as specific concerns regarding body parts, forms, or functions. The test is divided into two sections: BUT-A (34 items) and BUT-B (37 items). In line with previous validation studies, the BUT-A scores were combined into a global severity index (GSI) and subdivided into five subscales resulting from a factor analysis: fear of gaining weight (WP), body image concerns (BICs), body image-related avoidance (A), compulsive monitoring of physical appearance (CSM) and depersonalisation (D). The BUT-B scores were aggregated into two global measures (Positive Symptom Totality, PST, and Positive Symptom Distress Index, PSDI) and eight factors examining specific concerns for certain body parts or functions. Higher scores indicate greater body image-related distress.

#### 2.2.4. Short-Form Health Survey (SF-36)

The SF-36 is a generic quality of life assessment instrument designed to measure eight dimensions of health: physical functioning (PF, 10 items), role limitations caused by physical problems (RP, 4 items), bodily pain (BP, 2 items), general health perception (GH, 5 items), vitality (VT, 4 items), social functioning (SF, 2 items), role limitations caused by emotional problems (RLE, 3 items) and perceived mental health (MH, 5 items). In addition, the questionnaire includes a single item assessing the perceived change in general health status over the course of one year (health transition).

### 2.3. Statistical Analysis

Appropriate statistical tests were applied according to the nature of the data distribution. *T*-tests for independent samples or paired-sample *t*-tests were used in the case of the normality of the data distribution, while in cases where the data did not meet the normality criteria, the equivalent non-parametric tests, i.e., the Mann–Whitney U test and the Wilcoxon signed-rank test, were applied. Shapiro–Wilk tests were conducted to assess the normality of the variables, and the results of the normality tests were also confirmed visually using histograms. Exploratory, data-driven stepwise multiple regression with a forward method was used to identify potentially important predictors among a large set of baseline variables and their predictive value in a hypothesis-generating rather than conclusive manner. Although this method may be susceptible to Type I errors in general, considering the exploratory nature of the predictive analysis in this study and the large number of baseline variables, these regression analyses could suggest potential relationships that may have been intriguing for further investigations. Regarding the issue of missing values, different strategies have been used in general in the literature, including but not limited to omitting the participant with the missing data whatsoever, omitting the observation with the missing data in the analysis, or imputing the missing data with different methods. Imputation using the series mean was selected for its simplicity, transparency, and consistency with some previous exploratory clinical studies. It allowed us to avoid reducing the power further via listwise deletion, which would have been especially detrimental given the modest sample size. In order to address the issue of multiple comparisons, the False Discovery Rate (FDR) was used. Unlike more conservative methods, the FDR aims to limit the proportion of false positives, making it more powerful (i.e., less likely to miss true effects). Statistical analysis was conducted using SPSS software (25.0) (SPSS Inc., Chicago, IL, USA), with a significance level of 0.05.

## 3. Results

The results are presented initially for the entire sample of 97 patients and subsequently for the subsample of 33 patients undergoing follow-up. Sex differences will also be investigated for the total sample, as well as the sample with follow-up. The analyses examining the impact of the intervention on various dependent variables were performed exclusively on the subgroup of participants who completed the follow-up phase. At the end, the results of the exploratory regression analysis are presented.

Throughout the text, the following abbreviations will be used to refer to the assessments that have been conducted before and after the intervention: BMI for the body mass index, BDI-II for the Beck Depression Inventory—second edition, BES for the Binge Eating Scale, and BUT for the Body Uneasiness Test. The subscales of the BUT include the global severity index (BUT_GSI), the weight phobia subscale (BUT_WP), the body image concerns subscale (BUT_BIC), the avoidance subscale (BUT_AV) the compulsive monitoring subscale (BUT_CSM), the depersonalisation subscale (BUT_DEP), the BUT-B Positive Symptom Total Score (BUT_PST) and the BUT-B Positive Symptom Distress Index (BUT_PSDI). Measures of the SF-36 include the physical function (SF_PF), role limitations due to physical health (SF_RLP), role limitations due to emotional problems (SF_RLE), emotional well-being (SF_WELL), social function (SF_SF), and general health (SF_GH) scores.

### 3.1. Total Sample

The initial database comprised 97 patients. The means and standard deviations of the scores of all the measures and subscales broken down by sex are presented in Table 1. Out of the 97 patients, 25 were male, with a mean age of 41.68 years (SD = 10.58) and a mean education level of 10.45 years (SD = 3.01), while 72 were female, with a mean age of 44.05 years (SD = 11.59) and a mean education level of 10.59 years (SD = 3.66).

#### Sex Differences

The analysis of sex differences was conducted by applying the False Discovery Rate (FDR) correction to the results of the Mann–Whitney U test, to handle the problem of multiple comparisons. This analysis showed no significant differences between men and women in any of the scales considered. This indicates that, within this sample, no significant variations emerged between the two sexes in terms of BMI or psychological characteristics, such as levels of depression, uncontrolled eating episodes, or physical functioning.

### 3.2. Follow-Up Sample

Of all patients initially included in the database, 33 responded to the request to participate and completed the questionnaires in the follow-up phase, which took place an average of 311 days after surgery. Of these, 8 were men (mean age = 45.62 ± 9.13 years; schooling = 9.66 ± 2.58 years), and 25 were women (mean age = 43.48 ± 13.92 years; schooling = 11.81 ± 4.01 years). Table 2 shows the mean and standard deviations for all the scores and subscales of the assessments carried out before and after the intervention.

#### 3.2.1. Sex Differences

After correction for the False Discovery Rate (FDR), no significant differences emerged between the two sexes in any of the scales analysed. This indicates that men and women in the sample studied do not show any significant changes in BMI or psychological profile scores, including depression, uncontrolled eating, and physical functioning, both before and after the intervention. This result this may also be considered unsurprising given the limited statistical power of this specific comparison.

#### 3.2.2. Post- Versus Pre-Surgery in Males and Females Combined

The analysis of the pre- and postoperative assessments on the entire sample, performed by means of Wilcoxon’s signed-rank test and corrected for FDR, showed a significant decrease in BMI (Z = −5.012, *p* < 0.001, corrected, effect size = 0.616). In addition, a marginally significant decrease was found in BDI-II (Z = −2.525, *p =* 0.057, corrected, effect size = 0.310) and the BUT_BIC subscale (Z = −2.375, *p =* 0.068, effect size = 0.292), as well as a significant increase in the scores of BUT_PSDI (Z = −5.012, *p* < 0.001, effect size = 0.616), SF_PF (Z = −2.245, *p =* 0.073, effect size = 0.276), and SF_PAIN (Z = −2.597, *p =* 0.057, effect size = 0.319). The formula used to calculate the effect size is that proposed by [32], which defines it as the Z-value divided by the square root of the sample size in the independent samples and the total number of pairs in the paired samples. Following Tomczak and Tomczak (2014) [32], an effect size is considered to be very small if between 0 and 0.19, small between 0.20 and 0.49, medium between 0.50 and 0.79, and large if greater than 0.80.

#### 3.2.3. Post- Versus Pre-Assessments in Males and Females Separately

**Figure 1:**
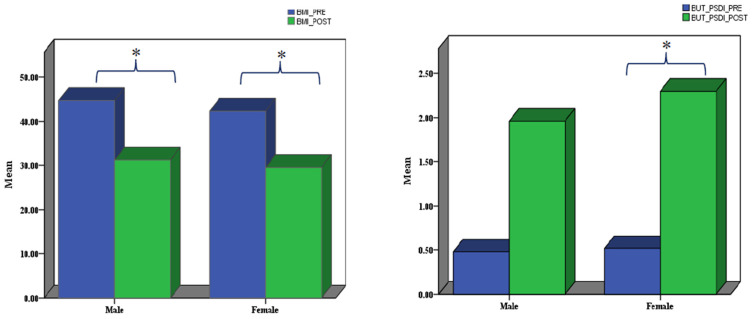
Variables showing significant changes after the surgery in males and females. Abbreviations: BMI = Bodymass index; BUT = Body uneasiness test; BUT_PSDI = Positive symptom distress index of BUT-B. * denotes a significant difference.

When the changes between pre- and post-surgery in the two sex groups were examined separately, different results emerged, with the exception of BMI, which was significantly reduced in both men (t(7) = 9.136, *p* < 0.001, corrected, effect size = 2.284) and women (Z = −4.372, *p* < 0.001, corrected, effect size = 0.618). It is important to emphasise that, in the female group, the non-parametric equivalent t-test for paired samples was applied, as the distribution of postoperative BMI did not meet the normality criteria. With regard to the other variables, in the male group there were no significant variations between the pre- and postoperative assessment. In contrast, in the female group, a significant change was observed in the BUT_PSDI subscale scores (Z = −4.372, *p* < 0.001, effect size = 0.618). This value did not show any sex differences in the initial assessment. Figure 1—Bar graphs representing the values of the psychological assessments for males and females separately.

### 3.3. Regression Results

**Figure 2:**
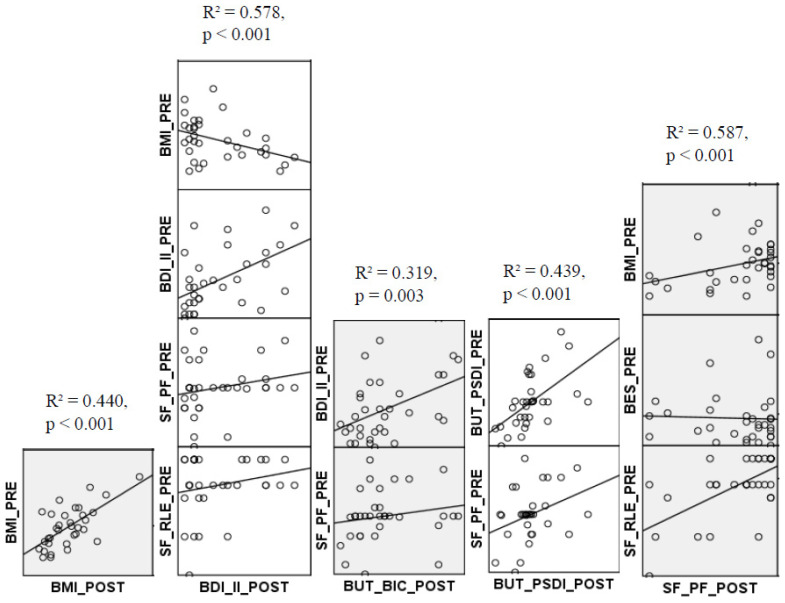
Trends between the all of these values and the predictors that resulted from running the regression analysis with each column representing a regression model consisting of the significant predictors. Abbreviations: BMI = Bodymass index; BDI_II = Beck depression inventory-second version; BES = Binge eating scale; BUT = Body uneasiness test; BUT_BIC = Body image concerns subscale of BUT-A; BUT_PSDI = Positive; symptom distress index of BUT-B. SF = 36-Item Short Form Survey (SF-36); SF_PF = Physical functioning subscale of SF; SF_RLP = Role limitation due to physical health subscale of SF; SF_RLE = Role limitation due to emotional problems subscale of SF.

To address the problem of multicollinearity, the Variance Inflation Factor (VIF), an index measuring the degree of correlation between independent variables, was calculated [33]. Multicollinearity occurs when the independent variables in a regression model are highly correlated, compromising their independence and making it difficult to assess the contribution of each in explaining variations in outcome. In the scientific literature, a VIF value between 1 and 5 is generally considered acceptable in regression analyses. A stepwise forward multiple regression analysis was conducted to identify the predictors of the BMI, BDI-II, BUT_BIC, BUT_PSDI, and SF_PF scores. The scatter plots in Figure 2 illustrate the trends between these values and the predictor variables used in the regression analysis.

#### 3.3.1. Prediction of Post-Surgery BMI

Regarding BMI, the only baseline variable that showed a predictive value was pre-surgical BMI, explaining 44% of the variance (R^2^ = 0.440, Adj. R^2^ = 0.422, F(1,32) = 24.342, *p* < 0.001, β = 0.695, *p* < 0.001, effect size = 0.785).

#### 3.3.2. Prediction of Post-Surgery BDI-II

Regarding the BDI-II scores after the intervention, the results of the multiple regression showed that a model consisting of BDI-II_PRE (β = 0.578, *p* < 0.001), SF_PF_PRE (β = 0.141, *p* = 0. 055), BMI_PRE (β = −0.419, *p* = 0.019), and SF_RLE_PRE (β = 0.092, *p* = 0.020) explained 57.8% of the variance in post-intervention BDI-II scores (R^2^ = 0.578, Adj. R^2^ = 0.518, F(4,32) = 9.59, *p* < 0.001, effect size = 1.369).

#### 3.3.3. Prediction of Post-Surgery BUT_BIC

Considering BUT_BIC_POST as the dependent variable, a model consisting of BDI-II_PRE (β = 0.081, *p* = 0.001) and SF_PF_PRE (β = 0.028, *p* = 0.042) was able to explain 31.9% of the changes in BUT_BIC scores after the intervention (R^2^ = 0.319, Adj. R^2^ = 0.274, F(2,32) = 7.040, *p* = 0.003, effect size = 0.468).

#### 3.3.4. Prediction of Post-Surgery BUT_PSDI

With regard to BUT_PSDI_POST as a dependent variable, a model consisting of BUT_PSDI_PRE (β = 2.124, *p* < 0.001) and SF_PF_PRE (β = 0.017, *p* = 0.019) explained 43.9% of the variations in BUT_PSDI scores after surgery (R^2^ = 0.439, Adj. R^2^ = 0.401, F(2,32) = 11.733, *p* < 0.001, effect size = 0.782).

#### 3.3.5. Prediction of Post-Surgery SF_PF

For the SF_PF_POST as a dependent variable, a model consisting of SF_RLE_PRE (β = 0.903, *p* < 0.001), BMI_PRE (β = 2.461, *p* = 0.002), and BES_PRE (β = 1.440, *p* = 0.009) explained 58.7% of the changes in SF_PF scores after the intervention (R^2^ = 0.587, Adj. R^2^ = 0.544, F(3,32) = 13.735, *p* < 0.001, effect size = 1.421).

## 4. Discussion

This retrospective and naturalistic study aimed to explore the psychological characteristics of individuals undergoing bariatric surgery and to identify possible predictors of surgical outcomes within a subgroup of participants who completed the follow-up assessment. The results indicate that, at baseline, there were no significant differences between males and females in psychological assessments in both the original and the reduced follow-up sample. Among those who participated in the follow-up, comparative analyses between pre- and post-surgical evaluations highlighted notable changes in certain parameters, such as a decrease in BMI and an increase in scores on the BUT_PSDI. The well-documented effectiveness of bariatric procedures in achieving BMI reduction [34] was corroborated by the present findings. Nonetheless, the observed improvements in psychological well-being were less substantial than those described in prior literature [35,36,37]. A possible explanation for this discrepancy could be the relative homogeneity of the sample with regard to concomitant psychological conditions such as depression or dysfunctional eating behaviour, which were already at relatively low levels at baseline.

In actual fact, the previously documented correlation between BMI and depression [37] did not emerge in either the baseline or the follow-up sample. However, follow-up participants showed a reduction in depressive symptoms after surgery, especially in subjects with higher pre-surgery BMI levels. This might suggest that a more pronounced change between pre- and post-surgery is associated with an improvement in emotional well-being. The post-surgical increase in PSDI scores represents a clinically relevant finding. The Body Uneasiness Test (BUT) serves as a tool to evaluate body-related experiences in clinical populations, encompassing various dimensions such as concerns about body image, compulsive behaviours related to physical appearance, and an overall index of severity [38]. In this study, the baseline scores for the BUT subscales were higher than normative values for non-clinical populations and aligned with those typically observed in individuals with obesity. However, contrary to previous findings [39], none of these subscales showed improvement following surgery. In fact, the PSDI score increased significantly after the intervention, indicating that body image distress may persist despite marked reductions in BMI. This outcome could be partially explained by the lack of a direct association between pre-surgical BMI and PSDI levels. Moreover, it is important to consider that this index may not be sufficiently responsive to notable BMI variations in clinical or psychotherapeutic contexts, either prior to or following surgical treatment.

This finding aligns with studies comparing patients who underwent cosmetic surgery following bariatric surgery with those who did not, highlighting the impact of additional surgical interventions on BUT scores [40]. Additionally, the absence of improvement in the other BUT subscales and the increase in PSDI are consistent with the findings of Rosta et al., who reported that body image perception is independent of BMI, emphasizing the need for psychological support alongside bariatric surgery to address body image concerns [41,42].

When analysing male and female subgroups separately, BMI showed a downward trend in both, in line with the literature on sex differences in bariatric surgery outcomes [43]. However, BUT_PSDI increased only in females, despite no significant differences at baseline between the two groups. This finding is expected, given the well-documented sex differences in body image concerns [44,45], and it suggests that males and females, despite similar baseline psychological assessments, may respond differently to the same intervention and experience distinct psychological outcomes.

Regarding predictors of post-surgical outcomes, previous studies have considered personality factors [46] and psychosocial predictors [47]. A meta-analysis by Livhits et al. (2012) reported that preoperative BMI, super-obesity, and personality disorders negatively influence surgical outcomes [48]. Other studies identified greater success in young female patients with high self-esteem, good mental health, high socioeconomic status, concerns about obesity, realistic expectations, and undisturbed eating behaviour [49]. In the present study, the only significant predictor of post-surgical BMI was pre-surgical BMI, explaining 44% of the variance, which aligns with previous research [50]. This finding underscores that, at least in the current sample, psychological characteristics did not predict intervention success, as measured by BMI reduction. In addition, it should be acknowledged that recent literature has highlighted that the psychological benefits of bariatric surgery may not be equally maintained over time in all patients. Several studies have shown that the initial improvement in psychological well-being observed after weight loss may diminish in the long term, especially in individuals with a pre-existing psychological vulnerability or maladaptive coping strategies [50], and depressive symptoms [51]. Furthermore, weight loss alone does not necessarily lead to a resolution of body image dissatisfaction, eating disorders, or emotional difficulties, which may persist or even emerge after surgery [42,52]. These results highlight the value of conducting thorough psychological evaluations prior to surgery and emphasise the necessity of a sustained psychological follow-up to enhance postoperative adjustment and mental well-being in patients undergoing bariatric procedures. Overall, while the findings point to a modest impact of bariatric surgery on patients’ psychological profiles, the regression analysis revealed valuable and potentially informative insights into the psychological condition of individuals after surgery. In terms of additional outcomes, the exploratory regression analysis revealed that a set of baseline variables—specifically BMI, pre-existing depressive symptoms, physical functioning, persisting concerns and distress related with body image and functioning, and role limitations caused by emotional problems—collectively predicted a depressive symptomatology following surgery. This pattern points to a potential link between patients’ preoperative physical functioning and emotional limitations and their depressive states after the intervention. Nonetheless, these associations should be interpreted with caution, considering that the post-surgical depressive symptoms generally remained below the threshold for clinically significant depression. As for body image distress, the postoperative PSDI scores were best predicted by a model that included preoperative PSDI levels and SF_PF scores, reinforcing the significance of physical functioning in shaping psychological responses after bariatric surgery.

## 5. Limitations

Despite the presence of a follow-up and the ecological validity typical of real-world clinical practice and a retrospective naturalistic study, the findings should be interpreted as hypothesis-generating rather than conclusive. The absence of a control group matched for weight and sex limits the possibility of drawing robust conclusions about the effects of the intervention. The longitudinal design of the study included a post-surgery follow-up; nevertheless, although the initial sample was quite consistent, the significant number of dropouts in the follow-up analysis may have significantly reduced the statistical power of the predictive analyses. A further limitation concerns the use of online assessment tools during the follow-up, which may have influenced the adherence to instructions and the quality of the collected data. It should also be emphasised that self-report measures in general are susceptible to the effects of response and memory bias and social desirability, which could have affected the results in an undesired manner. The high attrition rate and the geographical specificity regarding the studied sample should also be taken into account when interpreting the results. In addition, the decision not to focus on the presence of comorbid psychiatric disorders and its potential effects on the observed outcomes in the current study should be reminded to the readers. Altogether, although these factors do not completely invalidate the results, it is essential to take the exploratory nature of the study and the lack of experimental control, unmeasured confounders, and absence of patients’ drug regimen data into account for a correct interpretation of the data, since they can all limit the causal inferences.

## 6. Conclusions

Given the common use of pre-operative psychological evaluations as a screening method, more comprehensive studies incorporating a broader array of psychological and cognitive assessments may shed further light on treatment outcomes and the mental health of patients undergoing substantial life changes. These considerations can assist clinicians in better preparing patients for the postoperative journey and in offering more individualised support throughout the process. Moreover, investigating the psychological traits of bariatric surgery candidates and identifying predictors of treatment success could significantly advance the development of personalised interventions for obesity—an area of growing relevance within personalised medicine.

With the development of various therapeutic approaches, including metabolic bariatric surgery (MBS), endoscopic procedures, and drugs, it has become evident that individualised treatment is essential to optimise outcomes and minimise complications. The identification of specific psychological predictors allows the design of individualised treatment pathways, optimising multidisciplinary and psychological support at all stages of treatment, from pre-operative to follow-up.

## Figures and Tables

**Table 1:** Descriptive statistics regarding all the clinical assessments of the male, female, and total sample before surgery, out of whom a random number were assessed again after surgery.

	SEX	N	Mean	Std. Deviation	Std. Error Mean
BMI	M	25	43.0080	6.88076	1.37615
	F	72	40.8107	5.52220	0.65080
	Total	97	41.3770	5.94330	0.6035
AGE	M	25	41.6800	10.58584	2.11717
	F	72	44.0556	11.59272	1.36622
	Total	97	43.4433	11.33576	1.151
EDUCATION	M	25	10.4722	2.68483	0.53697
	F	72	10.5918	3.40035	0.40074
	Total	97	10.5610	3.21810	0.3267
BDI_II	M	25	7.4353	7.34159	1.46832
	F	72	10.7329	7.55669	0.89056
	Total	97	9.8830	7.72492	0.7843
BES	M	25	10.4611	10.01213	2.00243
	F	72	10.1952	7.40435	0.87261
	Total	97	10.2637	8.36638	0.8495
BUT_GSI	M	25	0.8953	1.00018	0.20004
	F	72	1.4796	1.25404	0.14779
	Total	97	1.3290	1.21620	0.1235
BUT_WP	M	25	1.0536	0.97950	0.19590
	F	72	1.7760	1.56574	0.18452
	Total	97	1.5898	1.47531	0.1498
BUT_BIC	M	25	1.2844	1.34374	0.26875
	F	72	1.9198	1.56194	0.18408
	Total	97	1.7560	1.52766	0.1551
BUT_AV	M	25	0.6133	0.95224	0.19045
	F	72	1.1088	1.25975	0.14846
	Total	97	0.9811	1.20326	0.1222
BUT_CSM	M	25	0.5200	0.61486	0.12297
	F	72	0.9722	0.93782	0.11052
	Total	97	0.8557	0.88572	0.0899
BUT_DEP	M	25	0.5520	1.04288	0.20858
	F	72	1.1889	1.28124	0.15100
	Total	97	1.0247	1.25075	0.127
BUT_PST	M	25	5.4400	6.54523	1.30905
	F	72	10.1389	8.21250	0.96785
	Total	97	8.9278	8.05353	0.8177
BUT_PSDI	M	25	0.4900	0.16991	0.03398
	F	72	0.5561	0.16744	0.01973
	Total	97	0.5391	0.16969	0.0172

Abbreviations: BMI = body mass index; BDI_II = Beck Depression Inventory—second version; BES = Binge Eating Scale; BUT = Body Uneasiness Test; BUT_GSI = global severity index of BUT-A; BUT_WP = weight phobia subscale of BUT-A; BUT_BIC = body image concerns subscale of BUT-A; BUT_AV = avoidance subscale of BUT-A; BUT_CSM = compulsive self-monitoring subscale of BUT-A; BUT_DEP = depersonalisation subscale of BUT-A; BUT_PST = Positive Symptom Total subscale of BUT-B; BUT_PSDI = Positive Symptom Distress Index of BUT-B.

**Table 2:** Mean and standard deviation of all the assessments before and after surgery of the sample on which the follow-up was conducted.

	N	Mean	Std. Deviation
MAX_WEIGHT	33	125.2727	22.76822
BMI_PRE (**)	33	42.8882	5.37253
BMI_POST	33	29.8901	5.62705
BDI_II_PRE	33	9.8125	7.79197
BDI_II_POST	33	7.0000	7.18505
BES_PRE	33	9.2188	8.44739
BES_POST	33	5.9697	4.59269
BUT_GSI_PRE	33	1.4875	1.08478
BUT_GSI_POST	33	1.2825	0.82448
BUT_WP_PRE	33	1.8164	1.37464
BUT_WP_POST	33	1.7917	0.96757
BUT_BIC_PRE	33	2.0337	1.39484
BUT_BIC_POST	33	1.4899	1.13188
BUT_AV_PRE	33	0.9949	1.06839
BUT_AV_POST	33	0.7323	0.90613
BUT_CSM_PRE	33	0.9293	0.76038
BUT_CSM_POST	33	0.8909	0.58329
BUT_DEP_PRE	33	0.9455	1.11722
BUT_DEP_POST	33	0.8838	0.82966
BUT_PST_PRE	33	10.1818	7.23902
BUT_PST_POST	33	13.7879	8.42525
BUT_PSDI_PRE (*)	33	0.5073	0.18500
BUT_PSDI_POST	33	2.2143	0.69847
SF_PF_PRE	33	60.5026	13.73800
SF_PF_POST	33	73.6364	31.55533
SF_RLP_PRE	33	66.6667	29.53635
SF_RLP_POST	33	81.8182	34.95126
SF_RLE_PRE	33	77.7773	25.45903
SF_RLE_POST	33	76.7677	38.62646
SF_ENERGY_PRE	33	60.4762	12.17738
SF_ENERGY_POST	33	66.5152	18.85325
SF_WELL_PRE	33	69.5619	11.62837
SF_WELL_POST	33	75.0303	17.94520
SF_SF_PRE	33	75.5952	18.08082
SF_SF_POST	33	68.0303	22.33934
SF_PAIN_PRE	33	64.8750	20.99549
SF_PAIN_POST	33	78.4848	26.10779
SF_GH_PRE	33	65.6548	13.28996
SF_GH_POST	33	67.1212	20.88025

Abbreviations: BMI *=* body mass index; BDI_II *=* Beck Depression Inventory—second version; BES *=* Binge Eating Scale; BUT *=* Body Uneasiness Test; BUT_GSI *=* global severity index of BUT-A; BUT_WP *=* weight phobia subscale of BUT-A; BUT_BIC = body image concerns subscale of BUT-A; BUT_AV *=* avoidance subscale of BUT-A; BUT_CSM *=* compulsive self-monitoring subscale of BUT-A; BUT_DEP *=* depersonalisation subscale of BUT-A; BUT_PST *=* Positive Symptom Total subscale of BUT-B; BUT_PSDI *=* Positive Symptom Distress Index of BUT-B; SF *=* 36-Item Short-Form Survey (SF-36); SF_PF *=* physical functioning subscale of SF; SF_RLP *=* role limitation due to physical health subscale of SF; SF_RLE *=* role limitation due to emotional problems subscale of SF; SF_WELL *=* emotional well-being subscale of SF; SF_SF *=* social functioning subscale of SF; SF_PAIN *=* pain subscale of the SF-36; SF_GH *=* general health subscale of SF. Note: (*) represents a significant difference from pre- to post-intervention with a *p*-value of <0.05, and (**) represent a significant difference with a *p*-value of < 0.001.

## Data Availability

The data presented in this study are available on request from the authors who reserve the right to make them available.
